# Structural Basis for DNA Recognition of a Single-stranded DNA-binding Protein from *Enterobacter* Phage Enc34

**DOI:** 10.1038/s41598-017-15774-y

**Published:** 2017-11-14

**Authors:** Elina Cernooka, Janis Rumnieks, Kaspars Tars, Andris Kazaks

**Affiliations:** 1 Latvian Biomedical Research and Study Centre, Riga, LV-1067 Latvia; 2 Faculty of Biology, Department of Molecular Biology, Riga, LV-1004 Latvia

## Abstract

Modern DNA sequencing capabilities have led to the discovery of a large number of new bacteriophage genomes, which are a rich source of novel proteins with an unidentified biological role. The genome of *Enterobacter cancerogenus* bacteriophage Enc34 contains several proteins of unknown function that are nevertheless conserved among distantly related phages. Here, we report the crystal structure of a conserved Enc34 replication protein ORF6 which contains a domain of unknown function DUF2815. Despite the low (~15%) sequence identity, the Enc34 ORF6 structurally resembles the gene 2.5 protein from bacteriophage T7, and likewise is a single-stranded DNA (ssDNA)-binding protein (SSB) that consists of a variation of the oligosaccharide/oligonucleotide-binding (OB)-fold and an unstructured C-terminal segment. We further report the crystal structure of a C-terminally truncated ORF6 in complex with an ssDNA oligonucleotide that reveals a DNA-binding mode involving two aromatic stacks and multiple electrostatic interactions, with implications for a common ssDNA recognition mechanism for all T7-type SSBs.

## Introduction

Since the very beginnings of molecular biology, bacteriophages have taken a prominent role as model systems for studying DNA replication. Due to the immense diversity of phage-encoded replication proteins and their associated host factors, phages arguably represent every theoretically possible replication mechanism^1^. Still, only a small number of model phages have been studied in substantial detail, and a comprehensive view of all known replication strategies remains to be established^2^. While recent advances in genome sequencing and annotation techniques have provided an unparalleled insight into the abundance and genetic diversity of bacteriophages in nature^3^, a major proportion of the phage-encoded proteins in the sequence data have remained ‘hypothetical’ as they lack any functional annotation due to negligible or undetectable sequence homology to genes with a known function^4^. In the vast array of the available sequences, a number of hypothetical proteins have been detected in phage DNA replication modules, which in many cases are conserved among distantly related species. Functional and structural studies of such proteins are of considerable interest as they can potentially uncover the function for an entire group of homologous proteins and provide a better understanding for phage replication mechanisms in general.

*Enterobacter cancerogenus* phage Enc34 is a characteristic member of the *Siphoviridae* family^5^, and is closely related to *Escherichia* phage Utah (GenBank ID: KY014601)^6^, *Staphylococcus* phage SA1 (GenBank ID: GU169904), *Providencia stuartii* phage RedJac (GenBank ID: JX296113)^7^, *Proteus mirabilis* phage pPM_01 (GenBank ID: KP063118), and *Salmonella* phages from the recently established *Chivirus* genus^8,9^. Similar genome size and high sequence conservation show that these phages constitute a distinct phylogenetic lineage with a peripheral relation to *Burkholderia* phages AH2 and BcepNazgul and *Xylella* phages Sano and Salvo, together with which they form a group of ‘*Nazgul-like* phages’^10^. The Enc34 replication module consists of a DNA primase, a putative transcription factor, a DNA polymerase, a virus-type replication-repair nuclease (VRR-NUC) domain protein, a DNA helicase, and four hypothetical proteins^5^. The hypothetical protein ORF6 of the Enc34 phage is conserved among all *Nazgul-like* phages and consists of a single domain, currently annotated as a domain of an unknown function DUF2815.

Here, we present structural and functional characterization of the Enc34 ORF6 that allows us to establish its function as a single-stranded DNA-binding protein (SSB) and extend this annotation to other DUF2815 family members. The SSBs are ubiquitous within all kingdoms of life and in DNA viruses, and protect ssDNA intermediates during replication, repair and recombination^2,11^. SSBs exhibit a pronounced affinity for ssDNA, and typically consist of one or more oligonucleotide/oligosaccharide-binding (OB)-fold domains^12,13^; in addition, the bacterial and phage SSBs contain a characteristic acidic C-terminal segment for interaction with other replication proteins^14^. Currently the only structurally characterized dsDNA phage SSBs are those of phages T4^15^, T7^16^, RB69^17^ and p2^18^, and no high-resolution structures in complex with DNA are known for any of them. In this study, we show that the Enc34 ORF6 is structurally homologous to the gene 2.5 protein from bacteriophage T7, and present a high-resolution structure of ORF6 in complex with ssDNA, which reveals for the first time the structural basis for the ssDNA-binding mechanism of T7-type SSBs.

## Results and Discussion

### Overall Structure

The structure of the full-length Enc34 ORF6 protein was solved to 1.50 Å resolution using a selenomethionine-substituted protein. The model contains a single protein chain with 179 modeled residues; the very first N-terminal and eleven C-terminal residues of the full-length protein were not visible in the electron density map due to disorder, and two loop regions (residues 39–43 and 98–100), were also poorly structured and therefore not included in the final model.

**Figure 1:**
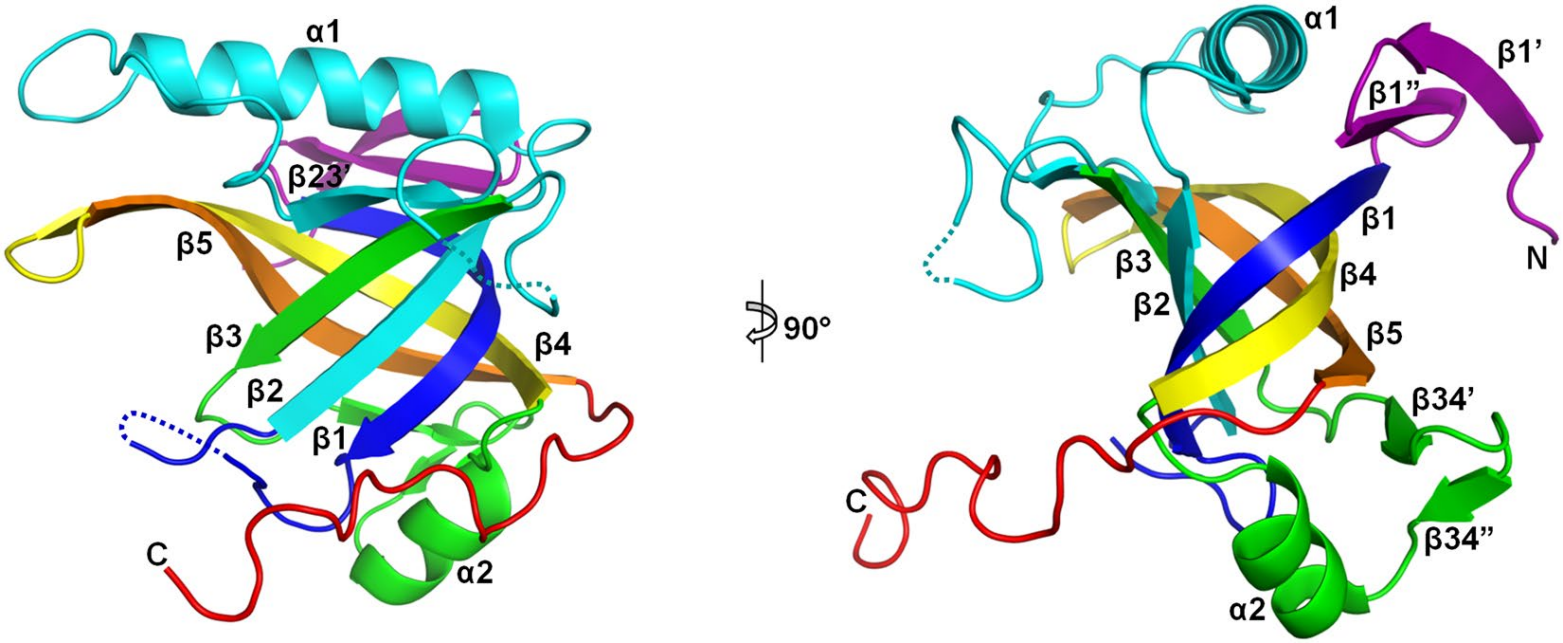
Three-dimensional structure of the phage Enc34 ORF6. The protein chain is rainbow-colored purple to red from the N- to the C-terminus. The conserved strands of the core β-barrel are named in accordance to Murzin^12^.

Apart from the C-terminus, the Enc34 ORF6 protein adopts a compact, roughly globular shape with a diameter of approximately 40 Å and has a mixed α/β architecture comprised predominantly of β-strands. The protein can be regarded as built up from a concave seven-stranded β-sheet at one side of the protein, of which the three central strands together with the two β-strands from the opposite side form a slightly flattened five-stranded β-barrel at the protein core, and two α-helices cap the β-barrel at both ends (Fig. 1). The core architecture of the protein clearly resembles the oligosaccharide/oligonucleotide-binding (OB)-fold, which is commonly defined as a five-stranded β-barrel with a capping α-helix^13^.

The ORF6 structure extends from the canonical OB-fold by having two extra strands (β1′ and β1″) at the N-terminus, a helix α1 and strand β23′ in the loop connecting β2 and β3, and two extra strands β34′ and β34″ between β3 and helix α2. The β23′ strand and the surrounding region form a V-shaped insertion between β3 and β5 that otherwise have only two hydrogen bonds between them, which further stabilizes the barrel. The helix α1 is rather long and packs with the three N-terminal strands, completely obstructing the opening of the β-barrel. The opposite side of the barrel is closed by the two short strands β34′ and β34″ and the OB-fold helix α2. All of the secondary structure elements share a common hydrophobic core and together form a single domain.

The C-terminus of the ORF6 protein is notably acidic with 16 of the 29 C-terminal residues being either aspartates or glutamates. These residues, as far as they are visible in the electron density map, do not form any secondary structure and extend away from the rest of the protein in a direction nearly parallel to strands β4 and β5. Interestingly, the very end of the traceable C-terminus inserts into the groove of the seven-stranded β-sheet of a neighboring molecule in the crystal, with possible implications for oligomerization, as discussed below.

### 3D Similarity to Other Proteins

While at the sequence level Enc34 ORF6 shows conservation with a number of recognizably homologous phage proteins from the DUF2815 family, its three-dimensional structure is similar to a wide variety of different OB-fold proteins. The structural homologs include, among others, a periplasmic copper-binding protein CusF, the N-terminal domain of the primosomal DNA replication protein PriB, and multiple single-stranded DNA-binding proteins (SSBs), which all have a β-barrel core similar to that of Enc34 ORF6. However, three structures currently in the Protein Data Bank (PDB) clearly form a high-similarity group with Enc34 ORF6, which includes two uncharacterized phage-related proteins from *Bacillus cereus* (PDB ID: 4JG2) and *Enterococcus faecalis* (PDB ID: 4KLK), and the bacteriophage T7 gene 2.5 protein (PDB ID: 1JE5), an extensively studied SSB. Of these, the phage-related *B*. *cereus* and *E*. *faecalis* proteins are closer relatives to Enc34 ORF6 with Cα atom root-mean-square deviations (RMSDs) of around 1.2 Å (over 129 and 114 residues, respectively), while the T7 gp2.5 has a Cα RMSD of about 1.55 Å (over 98 residues). The core β-barrel of the ORF6 protein (approximately 60 residues) also aligns reasonably well with numerous other SSBs and unrelated OB-fold proteins, in accordance with the known high level of structural conservation within this domain.

**Figure 2:**
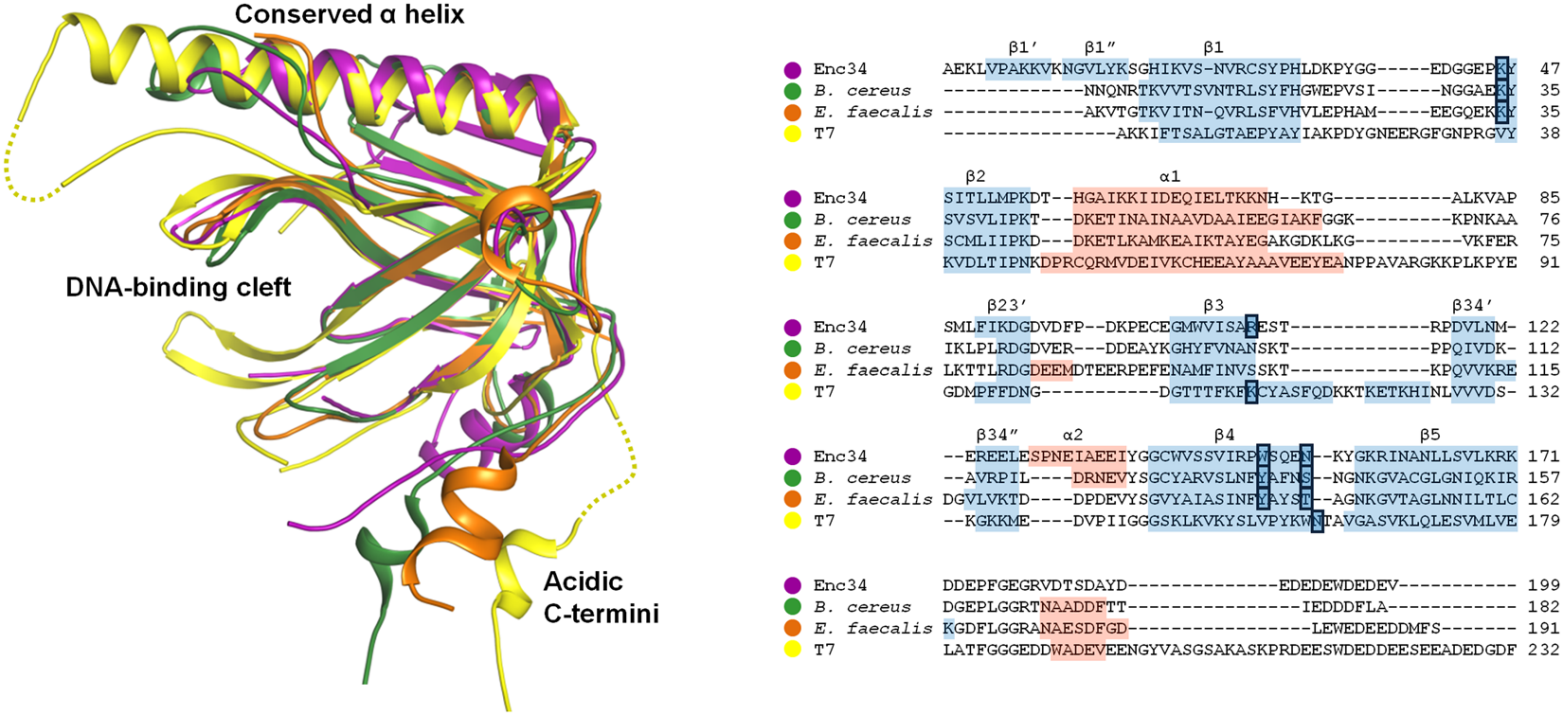
Structural alignment of the T7-type SSBs. The structures of the *Bacillus cereus* phage-related protein (green), *Enterococcus faecalis* phage-related protein (orange) and the T7 gp2.5 (yellow) are shown superimposed onto the Enc34 ORF6 (purple). A sequence alignment corresponding to the superimposition is presented on the right with conserved ssDNA-binding residues (see Fig. 6) boxed.

Previously, a high-sensitivity sequence analysis has suggested that the dsDNA phage-encoded SSBs fall into four major groups^2^. The T7 gp2.5 has been put forward as the class representative of a group of ‘T7-type’ SSBs, defined by sequence-based domain annotations PHA00458 and DUF2518^19^, and the high degree of 3D similarity clearly places the Enc34 ORF6 and the two phage-related proteins into this SSB group (Fig. 2). Of the other three recognized SSB types, only the *E*. *coli* SSB-like proteins still have a recognizable structural similarity to the T7-type, while the T4 gp32 group is too distinct for any sensible structural alignment, and no 3D structures of the N4-type SSBs are known as of now.

A hallmark feature that distinguishes T7-type SSBs from other OB-fold proteins is the long helix (α1 in ORF6 or αA in T7 gp2.5) immediately following strand β2, which caps one end of the β-barrel, whereas in the other structurally characterized SSBs the capping helix is markedly smaller and positioned between strands β3 and β4. Some features within the T7-type SSB group appear to be partially conserved such as a helix at the other end of the β-barrel which exists in ORF6 and the *B*. *cereus* protein, but not in the other two, while others are currently found in only a single representative, such as the two N-terminal β-strands in ORF6.

All four proteins also have a long acidic C-terminal tail, a characteristic component of all prokaryotic and bacteriophage SSBs, which in the T7 gp2.5 is implicated in interactions with the T7 DNA polymerase and primase-helicase, and has a proposed role in dimerization^20,21^. In the crystal, the C-termini of the Enc34 ORF6 protein lie amid the concave seven-stranded β-sheets of neighboring molecules along the crystallographic 2_1_ screw axis and bury a surface area of 842 Å^2^, which is the most extensive interface between the molecules. Notably, similar C-termini-mediated interactions are observed also in the crystal structures of both DUF2815 proteins and T7 gp2.5 (Supplementary Fig. S1), and could possibly account for the limited cooperativity observed in the gp2.5 binding to ssDNA^22^.

### DNA Binding Properties

**Figure 3:**
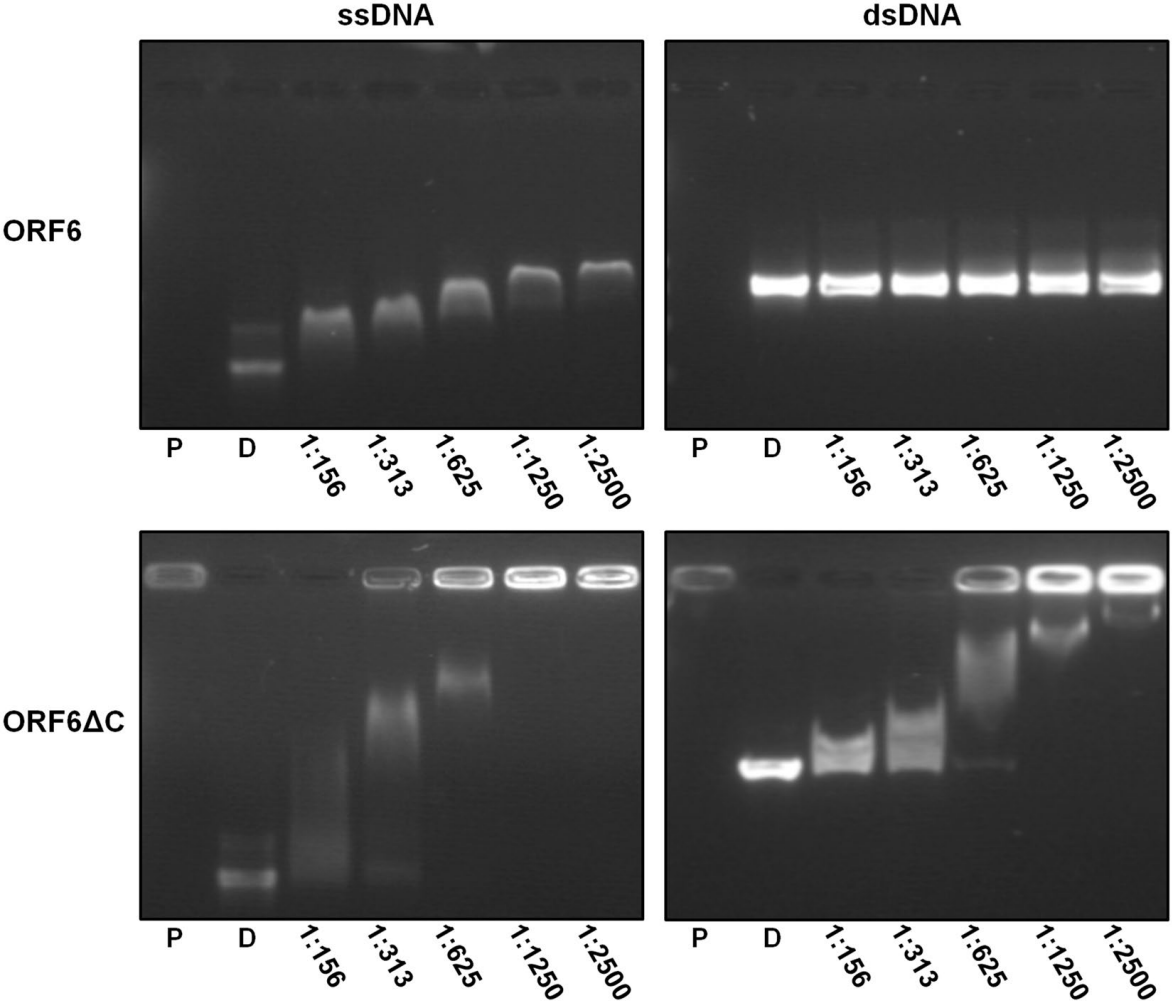
DNA binding properties of the wild-type and C-terminally truncated Enc34 ORF6 proteins. The DNA binding is demonstrated using electrophoretic mobility shift assays in four separate agarose gels. The assayed DNA-to-protein molar ratios are given below the respective tracks; P, protein only; D, DNA only. Full-length gels are presented in Supplementary Fig. S2.

To confirm that the Enc34 ORF6 indeed functions as an SSB, the protein was assayed in agarose gel electrophoresis in presence of different types of DNA. A clear mobility shift of the DNA was observed in presence of ssDNA (Fig. 3, top left), but not with dsDNA (Fig. 3, top right). In T7 gp2.5^23^ and T4 gp32^24^, the removal of the acidic C-terminus results in higher affinity for DNA, and to further test the DNA binding properties of the Enc34 ORF6, a recombinant protein ORF6ΔC was constructed with the C-terminal 21 residues lacking. While the ssDNA-binding affinity of the truncated protein remained unchanged (Fig. 3, bottom left), somewhat surprisingly, a similar affinity was observed also for dsDNA (Fig. 3, bottom right). T7 gp2.5 and T4 gp32 proteins bind dsDNA with a 10^4^-fold lower affinity than ssDNA^25^ which is consistent with our observations for the full-length ORF6 protein, but to our knowledge, the contribution of the C-terminal tail for the ssDNA-binding specificity has not been shown for other phage SSBs. Therefore, in ORF6, and possibly also other T7-type SSBs, the acidic C-terminal tail might play a role in allowing the protein to discriminate in favor of ssDNA.

### Structure of the ORF6-ssDNA Complex

**Figure 4:**
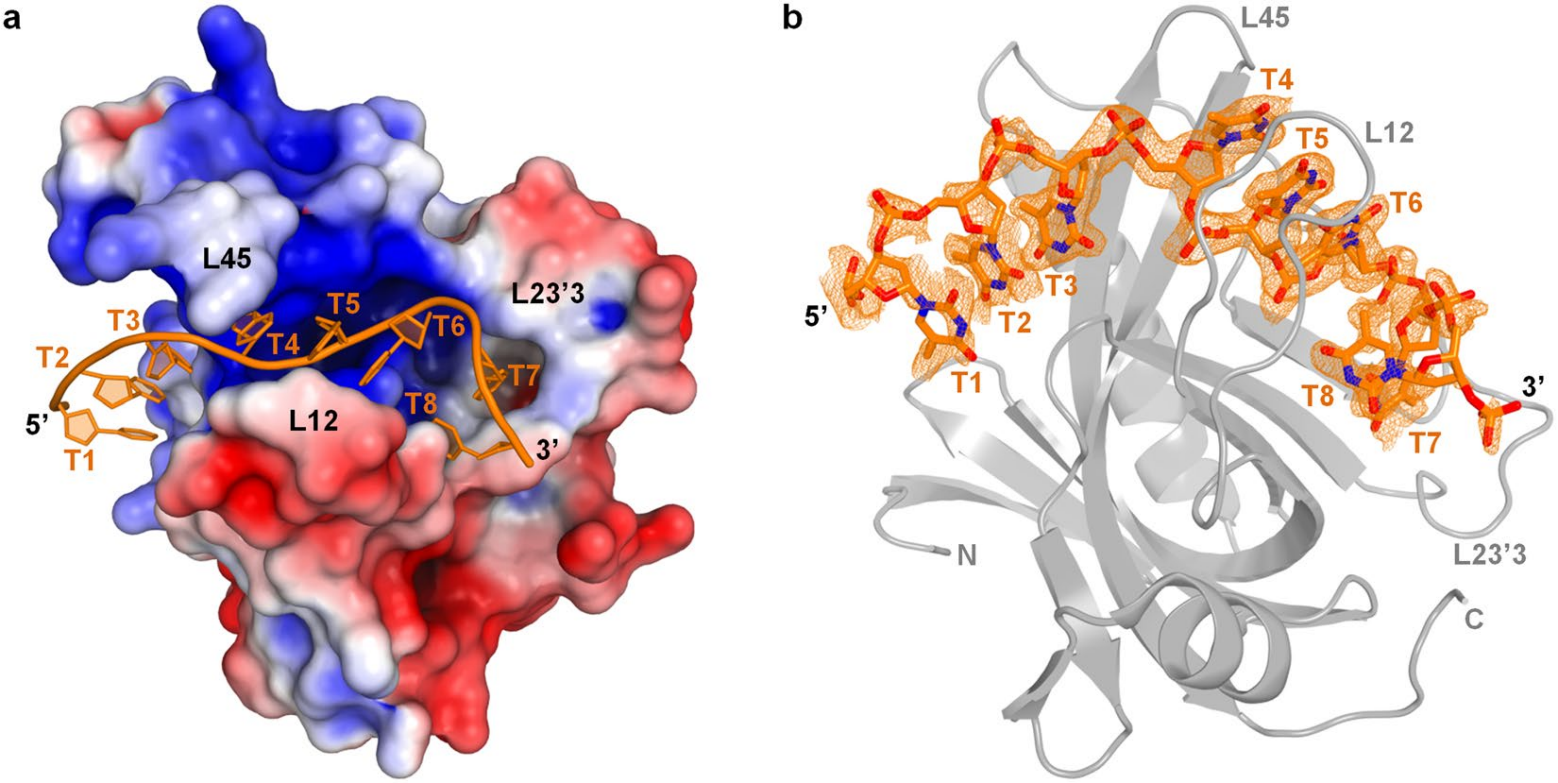
Overall structure of the ORF6ΔC-(dT)_35_ complex. (**a**) The DNA oligonucleotide (orange) binds to the positively charged cleft along the core OB-fold domain. (**b**) A different orientation of the complex with an overlay of a 2F_o_-F_c_ omit map of the DNA contoured at 0.7 σ. The thymidines are numbered T1-T8 in the 5′ to 3′ direction and the cleft-defining loops are indicated in both panels.

To further investigate the DNA-binding mechanism of the Enc34 ORF6, we determined the 3D structure of the protein in complex with ssDNA. While co-crystallization attempts using the full-length protein were unsuccessful, the truncated ORF6ΔC protein could be crystallized both in absence of DNA and in presence of oligo-thymidine (dT)_35_. ORF6ΔC crystals without bound DNA diffracted to a slightly higher resolution (1.34 Å) than those of the full-length protein and had three molecules in the asymmetric unit. Due to different packing of loops and N-termini in the crystal, the three protein chains have Cα RMSDs of 0.8 to 1.1 Å when superimposed with the full-length ORF6. The ORF6ΔC-(dT)_35_ crystals diffracted to 1.56 Å and contained a single chain of ORF6ΔC in the asymmetric unit bound to a stretch of nine thymidine nucleotides (Fig. 4). Successful crystallization of the complex presumably required a partial degradation of the DNA oligonucleotide as the crystals appeared only several months after the drops were set up.

The complex structure reveals the DNA strand stretching across the entire ligand-binding surface of the OB-fold domain, which is largely positively charged and enclosed by loops L12, L23′3 and L45 that connect strands β1 and β2, β23′ and β3, and β4 and β5, respectively (Fig. 4a). The interaction thus involves portions of all five strands of the β-barrel and buries a total of 891 Å^2^ of the solvent-accessible surface of the ORF6ΔC protein. Upon ssDNA binding, the core of the domain remains unchanged, but the cleft-defining loops L12 and L23′3 shift to accommodate the nucleic acid and, as a result, become ordered compared to the structures without bound DNA. Nucleotides T7 and T8 are less well defined in the electron density map, indicative of a certain flexibility at the 3′-end of the bound DNA, but the partial density of the sugar-phosphate backbone and the bases still allows them to be modeled. Lastly, the sugar and base moieties of T9 are not visible, and only the phosphate is included in the final model (Fig. 4b).

**Figure 5:**
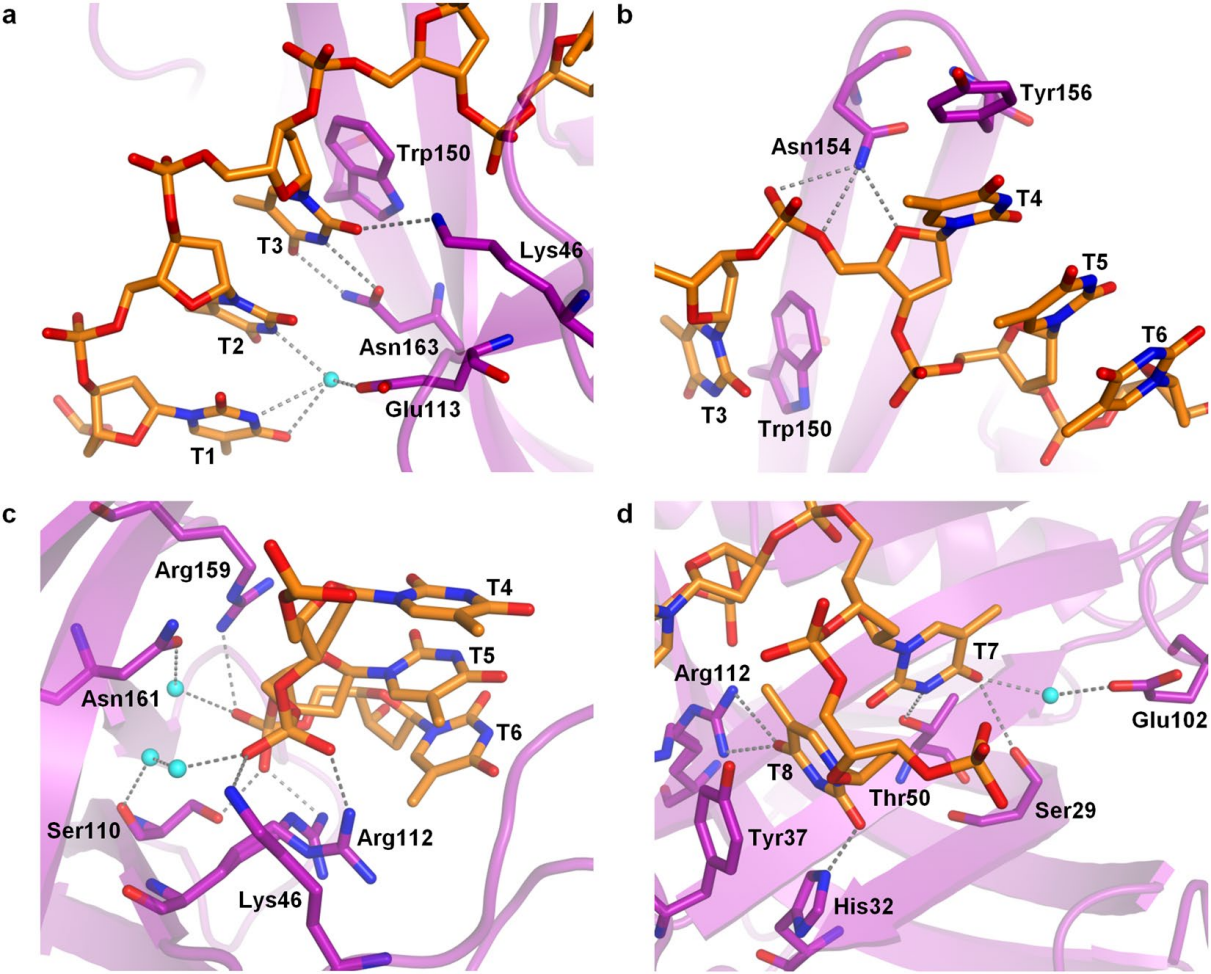
A detailed view of the ORF6-ssDNA interface. The interactions involving nucleotides T1 to T3 (**a**), T4 to T6 (**b**,**c**) and T7 to T8 (**d**) are shown. The ORF6 protein is shown in purple, the bound DNA in orange and the water molecules involved in hydrogen bonding in cyan.

The protein-DNA interface reveals an extensive network of interactions (Fig. 5) that involves both stacking of aromatic side chains with DNA bases and a number of hydrogen bonds and electrostatic interactions between the protein and the DNA. The three 5′-terminal bases (T1-T3) form a slightly slanted stack, which at the 3′ end is topped by the indole ring of Trp150. While the nucleotides T1 and T2 extend away from the binding cleft and contact the protein only through water-mediated hydrogen bonds, the T3 base faces inwards, stacks with the Trp150 side chain and forms three hydrogen bonds with polar residues from the core β-barrel and nearby loops (Fig. 5a). The T4 base stacks with the side chain of Tyr156, resulting in a prominent twist in the DNA backbone which causes the base to face away from the protein. The stack is further extended by bases T5 and T6 (Fig. 5b), while the backbone is clenched within the binding cleft by multiple direct and water-mediated hydrogen bonds and electrostatic interactions between the protein and the phosphate moieties. Notably, the side chain of Arg112 is observed in two alternative conformations in the crystal, in which it interacts with either the phosphate of T5 or that of T6 (Fig. 5c). Following the T4-T6 stack, the bases T7 and T8 are buried in-between loops L12 and L23′3, causing another bend in the sugar-phosphate backbone. Both nucleotides interact with the protein via base-specific hydrogen bonds and the T8 base additionally stacks with the side chain of Tyr37 (Fig. 5d). At the very 3′-end of the bound DNA, the T9 phosphate forms a single hydrogen bond with Tyr30 (not shown).

The multiple hydrogen bonds between the protein and the thymidine bases appear to suggest a certain sequence specificity for the interaction, and the affinity of the ORF6 protein for different nucleotide sequences has indeed not been determined. However, it can be noted that in a study where a single OB-fold domain from the telomere-end protection protein Pot1 was complexed to different ssDNA sequences, Dickey *et al*.^26^ showed that the combined flexibility of the nucleic acid and the protein allows a great variety of sequences to be bound with thermodynamic equality. Accordingly, it is likely that the structure of ORF6ΔC-(dT)_35_ represents one of several possible DNA-binding states which are nevertheless similar in the underlying interactions for DNA recognition.

### ssDNA Binding Among T7-type SSBs

**Figure 6:**
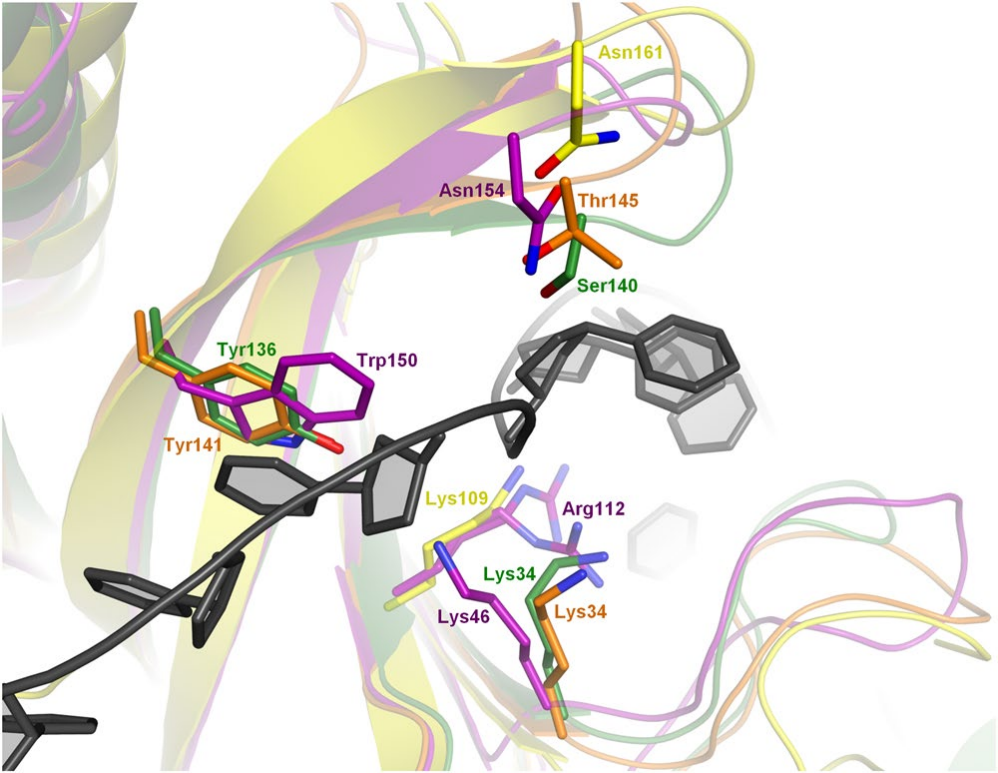
Conservation of the DNA-binding residues among the T7-type SSBs. The conserved residues are shown as sticks in the *Bacillus cereus* phage-related protein (green), *Enterococcus faecalis* phage-related protein (orange) and the T7 gp2.5 (yellow) superimposed onto the Enc34 ORF6ΔC (purple) bound to (dT)_35_ (black).

While the DNA-binding surface in the known structures of the T7-type SSBs is only partially conserved, the overall DNA recognition mode of all T7-type SSBs likely follows similar principles (Fig. 6). First, the stacking interaction at the 5′ terminus of the bound DNA appears to be clearly conserved. The Trp150 in ORF6 has corresponding aromatic residues Tyr136 and Tyr141 in the *B*. *cereus* and *E*. *faecalis* proteins, respectively (Fig. 2, right), whereas in the T7 gp2.5 a possible counterpart appears to be the Tyr158, which is likewise located at the N-terminus of the strand β4 and has also been experimentally shown to be necessary for ssDNA binding^23^. Next, a polar residue corresponding to Asn154 in ORF6 is found in all four structures as Ser140, Thr145, and Asn161 in the *B*. *cereus*, *E*. *faecalis* and T7 proteins, respectively. The residue functions in stabilizing the sharp turn in the DNA backbone which suggests that a similar kink is maintained in all T7-type SSBs. Finally, the electrostatic interactions within the DNA-binding cleft are also evidently conserved. A variety of basic residues are present within the narrow cleft, and of those a residue corresponding to Lys46 in ORF6 is conserved among all three DUF2815 proteins, and the Arg112 of ORF6 corresponds to Lys109 of gp2.5.

**Figure 7:**
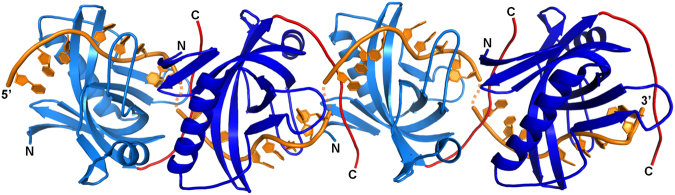
An oligomeric ssDNA-binding model of the Enc34 ORF6 protein. Four ORF6 molecules along the crystallographic 2_1_ axis are fitted with superimposed ssDNA molecules from the ORF6ΔC-(dT)_35_ complex. In the model, the protein monomers oligomerize via contacts between the C-termini (red) and the N-terminal strands of the OB-fold domain of the ORF6 protein (blue). The 3′ and 5′ ends of the fitted ssDNA oligonucleotides (orange) come in close vicinity to those of the neighboring complexes and could form a continuous strand.

Most of the currently known high-resolution SSB-ssDNA complex structures consist of an assembly of four OB-fold domains that form a functional unit for DNA binding^27–31^. In contrast, T7 gp2.5 is thought to bind DNA as a monomer or a dimer^16^. Size-exclusion chromatography of the Enc34 ORF6 protein suggests that it exists as a monomer or possibly as a transient dimer in solution (data not shown), although the second capping helix α2 in ORF6 would prevent its dimerization akin to T7 gp2.5. While the oligomeric state of ssDNA-bound Enc34 ORF6 remains unknown, the rather extensive C-termini-mediated interactions among neighboring molecules in the crystal provide an intriguing clue for possible higher-order assembly, which would allow for contiguous binding of an ssDNA sequence in a roughly helical conformation (Fig. 7). Such model would infer a functional similarity between the Enc34 ORF6 and the T4 gp32 and the SSB from *Sulfolobus solfataricus*, which both are thought to bind DNA as monomers in a contiguous fashion^32,33^, however, further experimental evidence is required to determine the biological significance of the assembly.

## Methods

### Construction of Plasmids

The coding sequence for the full-length ORF6 protein (199 residues) was PCR-amplified from Enc34 genomic DNA (GenBank ID: JQ340774) using a forward primer 5′-TGGATCCGGAAAACCTGTATTTCCAAGGCCTCGCAGAGAAATTAGTCCCAGC-3′ to introduce an N-terminal TEV protease cleavage site, and a reverse primer 5′-GTGCTTAAGTTAAACTTCGTCTTCGTCCCAC-3′. The amplified DNA was digested with *Bam*HI and *Bsp*TI (sites underlined) and cloned into a pETDuet-1 vector (Novagen) in-frame with an N-terminal 6xHis-tag, resulting in plasmid pETDt_Enc_ORF6. To construct a plasmid encoding a C-terminally truncated ORF6ΔC protein (residues 1–178) a DNA fragment was amplified from pETDt_Enc_ORF6 using the same forward primer and a reverse primer 5′-TATCTTAAGTTATTCACCGAATGGCTCGTCGTC-3′, which was likewise *Bam*HI/*Bsp*TI-cloned into pETDuet-1 to yield plasmid pETDt_Enc_ORF6ΔC.

### Protein Expression and Purification

To produce the ORF6 and ORF6ΔC proteins, pETDt_Enc_ORF6 or pETDt_Enc_ORF6ΔC were transformed into *Escherichia coli* BL21(DE3) cells and the bacteria were grown in 2xTY medium supplemented with 50 μg/mL ampicillin. Both ORF6 and ORF6ΔC were only partially soluble when produced at 37 °C, but the solubility significantly increased when the expression was performed at lowered temperatures. To obtain protein for purification, the culture was grown at 25 °C until its OD_600_ reached 0.6–0.8, after which the growth temperature was further reduced to 22 °C and IPTG was added to a final concentration of 0.01 mM to induce protein expression. Following overnight incubation, the cells were harvested by centrifugation, resuspended in the lysis buffer (20 mM Tris-HCl pH 8.0, 300 mM NaCl) and disrupted by sonication. The cell lysate was clarified by centrifugation and manually applied onto a 1 mL HisTrap FF crude column (GE Healthcare) for affinity purification. The column was washed with lysis buffer containing 20 mM imidazole, and the bound target protein was eluted with an equivalent buffer containing 300 mM imidazole. The eluted protein was digested overnight with recombinant TEV protease at 4 °C in presence of 1 mM DTT. The preparation was applied to a 5 mL HiTrap Desalting column (GE Healthcare) pre-equilibrated with the lysis buffer to remove the imidazole, followed by another round of nickel-affinity chromatography. The flow-through containing the cleaved target protein was diluted with 20 mM Tris-HCl pH 8.0 to reduce NaCl concentration to 100 mM and loaded onto a 1 ml MonoQ 5/50 GL column (GE Healthcare) using an ÄKTA FPLC chromatography system (Amersham Biosciences). The bound proteins were eluted with a linear gradient of 0.1 to 1 M NaCl in 20 mM Tris-HCl pH 8.0, and the fractions containing the purified ORF6 or ORF6ΔC proteins were pooled and used for crystallization or electrophoretic mobility shift assays.

To produce selenomethionine-substituted ORF6, *E*. *coli* B834(DE3) cells containing pETDt_Enc_ORF6 were grown in 2xTY medium at 25 °C until OD_600_ of the culture reached 0.8–1.0. The cells were then centrifuged, resuspended and incubated in SelenoMet™ Medium Base supplemented with SelenoMet™ Nutrient Mix (Molecular Dimensions) at 25 °C for 2 h. Thereafter, 1x SelenoMethionine solution and 0.1 mM IPTG were added and the cultivation was continued overnight at 25 °C. The protein was extracted and purified following the same protocol as for the native ORF6, except that 5 mM DTT was added to the lysis buffer and all other purification buffers contained 1 mM DTT.

### Crystallization and Data Collection

Prior to crystallization, SeMet-ORF6 and ORF6ΔC proteins were concentrated to 10 mg/mL using Amicon 10 kDa MWCO filters (Millipore) and crystallized using the sitting-drop vapor-diffusion technique. The SeMet-ORF6 crystals used for data collection were obtained by mixing 1 μL of the concentrated protein solution with 1 μL of a solution containing 0.1 M Bis-tris methane pH 5.5, 0.3 M MgCl_2_, 26% PEG 3350 and 1 mM DTT. Crystals of the ORF6ΔC protein were grown in a solution containing 0.04 M potassium dihydrogen phosphate, 16% PEG 8000 and 20% glycerol. To obtain the ORF6ΔC-(dT)_35_ complex, the protein and DNA oligonucleotide (Microsynth AG) were mixed prior to crystallization at a molar ratio of 1:2 (final concentrations 10 mg/mL and 11 mg/mL, respectively). Crystals of the complex appeared after two months in conditions containing 0.1 M HEPES pH 7.0 and 30% Jeffamine ED-2001. For data collection, the crystals were flash-frozen in liquid nitrogen. Prior to vitrification, the crystals of SeMet-ORF6 and the ORF6ΔC-(dT)_35_ complex were transferred to a mother liquor containing 30% glycerol; the ORF6ΔC crystals were used without additional cryoprotectant. Diffraction data from SeMet-ORF6 and ORF6ΔC crystals were collected at MAX-lab beamline I911–3 (Lund University, Sweden) and for the ORF6ΔC-(dT)_35_ complex at BESSY II beamline 14.1 (Helmholtz-Zentrum Berlin, Germany).

### Structure Determination and Analysis

The diffraction images were processed with MOSFLM^34^ and scaled using SCALA^35^ from the CCP4 suite^36^. The selenium sites were located and initial phasing was done in SHELX C/D/E^37^, followed by density modification in DM^38^. The initial model was generated by auto-tracing the protein chain in BUCCANEER^39^, followed by manual re-building in COOT^40^. Structure of the ORF6ΔC protein was solved by molecular replacement in MOLREP^41^ using SeMet-ORF6 coordinates without the 21 C-terminal residues as the search model. The ORF6ΔC-(dT)_35_ complex structure was solved using PHASER^42^ with the ORF6ΔC protein as the search model. Inspection of the resulting map clearly revealed extra electron density close to the presumed DNA-binding surface of the protein, and the oligonucleotide was modeled using the DNA building functionality in COOT. All models were refined using REFMAC^43^ and validated in COOT and MolProbity^44^. Data collection, scaling and refinement statistics are given in Table 1.

**Table 1:** Crystallographic data collection, scaling, refinement and model validation statistics.

	ORF6	ORF6ΔC	ORF6ΔC-(dT)_35_
**Data collection and scaling**			
Beamline	MAX-lab I911-3	MAX-lab I911-3	BESSY II 14.1
Space group	P2_1_2_1_2_1_	P2_1_	P6_1_
Cell parameters			
a, b, c (Å)	38.08, 67.20, 76.83	50.77, 103.62, 59.29	43.49, 43.49, 179.11
β (°)	90.00	111.09	90.00
Wavelength (Å)	0.97671	0.97181	0.91840
Resolution (Å)	34.1–1.50	30.9–1.34	59.7–1.56
Highest resolution bin (Å)	1.58–1.50	1.42–1.34	1.64–1.56
R_merge_	0.104 (0.770)	0.047 (0.369)	0.071 (0.679)
Total number of observations	115646	386968	129354
Number of unique reflections	32072	126056	27151
I/σI	6.9 (2.5)	12.6 (2.8)	9.5 (2.1)
Completeness (%)	99.4 (100.0)	99.3 (99.1)	99.7 (99.7)
Multiplicity	3.6 (3.6)	3.1 (2.7)	4.8 (4.7)
**Refinement**			
R_work_	0.157	0.150	0.141
R_free_	0.193	0.186	0.191
Average B factor (Å^2^)			
Protein	23.4	21.3	26.7
DNA			55.7
From Wilson plot	13.7	12.4	20.2
Number of atoms			
Protein	1427	4176	1444
DNA			164
Water	170	530	137
Other	2	17	25
RMSD			
Bong lengths (Å)	0.012	0.011	0.011
Bond angles (°)	1.571	1.627	1.523
Ramachandran plot			
Residues in favored regions (%)	98.3	98.8	98.3
Residues in allowed regions (%)	100.0	100.0	100.0

Values in parentheses are given for the highest resolution bin. RMSD, root-mean-square deviation.

The structures were visualized and analyzed with PyMOL^45^. Structural homologs of the ORF6 protein were identified using the Dali server^46^. Subsequent superimpositions and RMSD calculations were done in LSQMAN^47^, in some cases preceded by SSM-superposition in COOT. The RMSD values were calculated using superimposed Cα atoms with a distance cutoff of 3.5 Å; the number of superimposed atoms were 129 (PDB ID: 4KLK), 114 (PDB ID: 4JG2), 98 (PDB ID: 1JE5), 68 (PDB ID: 2VB3), 62 (PDB ID: 1S3O), 62 (PDB ID: 1Z9F), 61 (PDB ID: 4GS3), 60 (PDB ID: 3TGY), 59 (PDB ID: 3LGJ) and 58 (PDB ID: 3VDY). An initial multiple sequence alignment of the Enc34 ORF6, *B*. *cereus* phage-related protein, *E*. *faecalis* uncharacterized protein and T7 gp2.5 (UniProt IDs: H6WYG2, Q73EI2, Q838W1, P03696) was generated using Clustal Omega^48^ and manually adjusted by examining the superimposed 3D structures in COOT. The protein-DNA and protein-protein interface areas were calculated in PISA^49^. The electrostatic surface of the ORF6 protein was calculated using PDB2PQR^50^ and APBS^51^ via the APBSTools2 plug-in in PyMOL and is represented at levels −3.5 to +3.5 in dimensionless units of k_b_ T e_c_^−1^, where k_b_ is Boltzmann’s constant, T is the temperature used in the calculation (310 K) and e_c_ is the charge of an electron.

### Electrophoretic Mobility Shift Assay (EMSA)

For the binding assays, an ssDNA fragment (~750 nt) was prepared from Enc34 genomic DNA using 60 cycles of asymmetric PCR with a 100-fold excess of the forward primer 5′-CTCACAAGGTATCTTAGAAACTATC-3′ over the reverse primer 5′-GTGCTTAAGTTACGAGTACCTTTTGACGGC-3′, and an equivalent dsDNA fragment was obtained by conventional PCR using the same primers. The PCR products were gel purified using the GeneJET Gel Extraction Kit (Thermo Fisher Scientific) and the DNA concentration determined spectrophotometrically. For the binding reactions, 5 μL of DNA at a concentration of 140 nM were mixed with 5 μL samples of ORF6 or ORF6ΔC proteins, prepared as two-fold serial dilutions from 350 μM stocks to yield molar excess ratios ranging from 1:156 to 1:2500. The reactions were incubated at room temperature for 20 minutes, loaded onto a 1% agarose gel containing ethidium bromide and run at 10 V/cm for 20 min in 1x TAE buffer, with subsequent DNA visualization under UV light.

### Data availability

Coordinates and structure factors for the reported structures are available from the Protein Data Bank with accession codes 5ODJ (SeMet-ORF6), 5ODK (ORF6ΔC) and 5ODL (ORF6ΔC-(dT)_35_ complex). Any other datasets supporting the conclusions of this article are available from the corresponding authors on reasonable request.

## Electronic supplementary material

Supplementary Figures S1 and S2
