# Supplementary material for: Structural Basis for DNA Recognition of a Single-stranded DNA-binding Protein from Enterobacter Phage Enc34
Source: Sci Rep. 2017 Nov 14;7:15529. doi: 10.1038/s41598-017-15774-y (PMC5686142; doi:10.1038/s41598-017-15774-y)
Supplement: Supplementary file 1 — Supplementary Figures S1 and S2 [file 41598_2017_15774_MOESM1_ESM.pdf]

Supplementary Information

Structural Basis for DNA Recognition of a Single-stranded DNA-binding Protein from *Enterobacter* Phage Enc34

Elina Cernooka, Janis Rumnieks, Kaspars Tars, Andris Kazaks

a

b

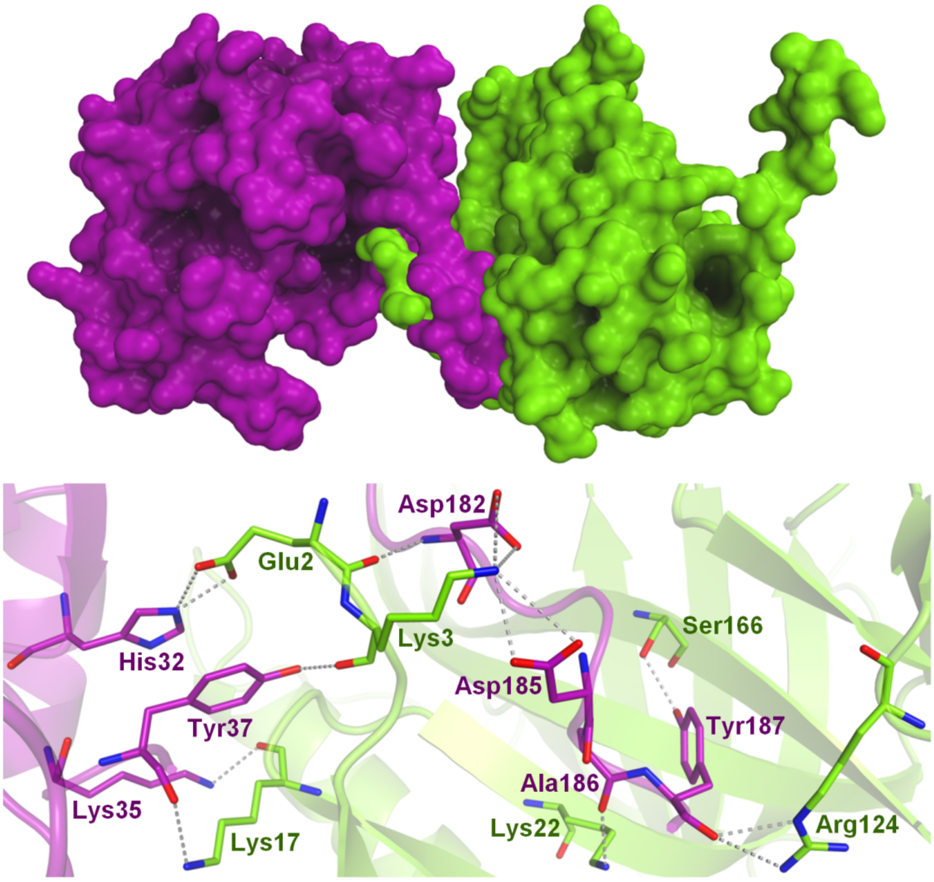

c

```
Enc34            AEKLVPAKKVKNGVLYKSGHIKVS-NVRCSYPHLDKPYGG-----EDGGEPKY   47
B. cereus        -------------NNQNRTKVVTSVNTRLSYFHGWEPVSI------NGGAEKY   35
E. faecalis      -------------AKVTGTKVITN-QVRLSFVHVLEPHAM-----EEGQEKKY   35
T7               ----------------AKKIFTSALGTAEPYAYIAKPDYGNEERGFGNPRGVY   38

Enc34            SITLLMPKDT--HGAIKKIIDEQIELTKKNH--KTG-----------ALKVAP   85
B. cereus        SVSVLIPKT---DKETINAINAAVDAAIEEGIAKFGGK---------KPNKAA   76
E. faecalis      SCMLIIPKD---DKETLKAMKEAIKTAYEGAKGDKLKG----------VKFER   75
T7               KVDLTIPNKDPRCQRMVDEIVKCHEEAYAAAVEEYEANPPAVARGKKPLKPYE   91

Enc34            SMLFIKDGDVDFP--DKPECEGMWVISAREST-------------RPDVLNM-  122
B. cereus        IKLPLRDGDVER---DDEAYKGHYFVNANSKT-------------PPQIVDK-  112
E. faecalis      LKTTLRDGDEEMDTEERPEFENAMFINVSSKT-------------KPQVVKRE 115
T7               GDMPFFDNG-----------DGTTTFKFKCYASFQDKKTKETKHINLVVVDS- 132

Enc34            --EREELESPNEIAEEIYGGCWVSSVIRPWSQEN--KYGKRINANLLSVLKRK 171
B. cereus        --AVRPIL----DRNEVYSGCYARVSLNFYAFNS--NGNKGVACGLGNIQKIR 157
E. faecalis      DGVLVKTD----DPDEVYSGVYAIASINFYAYST--AGNKGVTAGLNNILTLC 162
T7               --KGKKME----DVPIIGGGSKLKVKYSLVPYKWNTAVGASVKLQLESVMLVE 179

Enc34            DDEPFGEGRVDTSDAYD-------------EDEDEWDEDEV----------- 199
B. cereus        DGEPLGGRTNAADDFTT----------------IEDDDFLA----------- 182
E. faecalis      KGDFLGGRANAESDFGD----------------LEWEDEEDDMFS------- 191
T7               LATFGGGEDDWADEVEENGYVASGSAKASKPRDEESWDEDDEESEEADEDGDF 232
```

Supplementary Figure S1. Interactions between symmetry-related ORF6 monomers in the crystal and their conservation among the T7-type SSBs. (**a**) An overall view of two neighboring ORF6 molecules along the crystallographic 2_1 axis. The major intermolecular interface between the molecules that involves the C-terminus of one monomer (purple) and the β-sheet of a neighboring molecule (green) is visible in the forefront. (**b**) A close-up view of the hydrogen bonding and electrostatic interactions in the crystal contact. (**c**) A structure-based alignment of T7-type SSB sequences with residues involved in oligomerization highlighted in purple and green according to the monomer coloring in (**a**); hydrogen bond-forming residues are indicated with white letters, and salt bridge-forming residues with yellow.

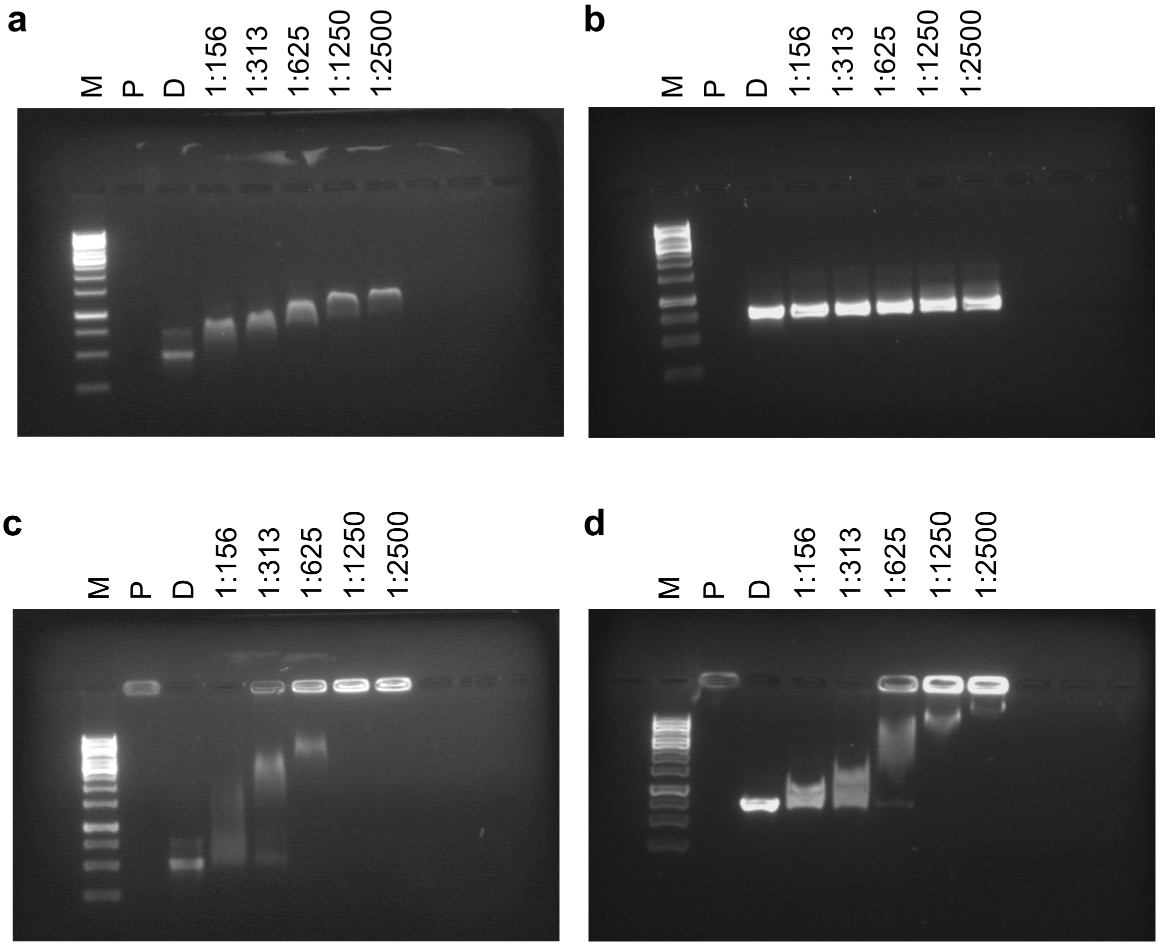

Supplementary Figure S2. DNA binding properties of the wild-type and C-terminally truncated (ΔC) Enc34 ORF6 proteins. The DNA binding is demonstrated using electrophoretic mobility shift assays in four separate agarose gels. (**a**), ORF6 + single-stranded DNA; (**b**), ORF6 + double-stranded DNA, (**c**), ORF6ΔC + single-stranded DNA, (**d**), ORF6ΔC + double-stranded DNA. Experimental details are given in the Methods section of the main article. The molar DNA-to-protein ratios tested in the experiment are given above the respective tracks; M, DNA marker (GeneRuler 1 kb, Thermo Scientific); P, protein only; D, DNA only.
